# Synthesis and Thermal Analysis of Non-Covalent PS-*b*-SC-*b*-P2VP Triblock Terpolymers via Polylactide Stereocomplexation

**DOI:** 10.3390/polym14122431

**Published:** 2022-06-15

**Authors:** Ameen Arkanji, Viko Ladelta, Konstantinos Ntetsikas, Nikos Hadjichristidis

**Affiliations:** Polymer Synthesis Laboratory, KAUST Catalysis Center, Physical Sciences and Engineering Division, King Abdullah University of Science and Technology (KAUST), Thuwal 23955, Saudi Arabia; ameen.arkanji@kaust.edu.sa

**Keywords:** polylactides, stereocomplexation, anionic polymerization, ring-opening polymerization, triblock terpolymers

## Abstract

Polylactides (PLAs) are thermoplastic materials known for their wide range of applications. Moreover, the equimolar mixtures of poly(L-Lactide) (PLLA) and poly(D-Lactide) (PDLA) can form stereocomplexes (SCs), which leads to the formation of new non-covalent complex macromolecular architectures. In this work, we report the synthesis and characterization of non-covalent triblock terpolymers of polystyrene-*b*-stereocomplex PLA-*b*-poly(2-vinylpyridine) (PS-*b*-SC-*b*-P2VP). Well-defined *ω*-hydroxy-PS and P2VP were synthesized by “living” anionic polymerization high-vacuum techniques with *sec*-BuLi as initiator, followed by termination with ethylene oxide. The resulting PS-OH and P2VP-OH were used as macroinitiators for the ring-opening polymerization (ROP) of DLA and LLA with Sn(Oct)_2_ as a catalyst to afford PS-*b*-PDLA and P2VP-*b*-PLLA, respectively. SC formation was achieved by mixing PS-*b*-PDLA and P2VP-*b*-PLLA chloroform solutions containing equimolar PLAs segments, followed by precipitation into *n*-hexane. The molecular characteristics of the resulting block copolymers (BCPs) were determined by ^1^H NMR, size exclusion chromatography, and Fourier-transform infrared spectroscopy. The formation of PS-*b*-SC-*b*-P2VP and the effect of molecular weight variation of PLA blocks on the resulting polymers, were investigated by differential scanning calorimetry, X-ray powder diffraction, and circular dichroism spectroscopies.

## 1. Introduction

Among synthetic aliphatic polyesters, poly(lactic acids)/polylactides (PLAs) have attracted enormous interest because they are biocompatible and biodegradable [1,2,3]. PLAs are derived from natural renewable resources, are non-toxic to the human body, and possess good thermomechanical properties [4,5]. As a result, PLAs have been widely used in a broad range of applications, such as packaging materials [6,7] and biomedical materials (e.g., surgical sutures, implant materials, and controllable drug delivery systems) [8,9,10], among many other applications [11,12,13,14].

PLAs also possess several inherent defects [15,16], such as long degradation periods and slow crystallization rates, leading to inferior properties, and low heat distortion temperature, which increases the difficulty of processing [17,18]. Diverse strategies and chemical modifications have been employed to overcome such drawbacks, including the use of blends and additives [19]. Another approach is the use of PLA stereocomplex (SC)-based copolymers to enhance the properties of PLA.

Lactide (LA) exists in two optically active forms, i.e., stereoisomers, (R,R)L-lactide (LLA) and (S,S)D-lactide (DLA), and one optically inactive form, (R,S)*meso*-lactide (*m*LA) [20,21]. When LA is converted into a polymer, the stereoregularity of the chain has a strong influence on the thermomechanical properties. Consequently, PLA derived from different stereoisomers exhibits various physical and chemical properties. For example, isotactic PLLA and PDLA with high stereoregularity are semi-crystalline polymers with a melting temperature (*T_m_*) between 170 and 190 °C [22], whereas atactic poly(*rac*-lactide) (PDLA) is amorphous due to the absence of stereoregularity. Moreover, the mixture of isotactic PLLA and PDLA can form superior material called PLA stereocomplex (SC).

The formation of SC crystallites between PLLA and PDLA was first reported by Ikada et al. in 1986 [23]. They found that an equimolar mixture of isotactic PLLA and PDLA in dichloromethane undergoes stereoselective physical association through multicenter hydrogen bonding interaction between the methyl and the carbonyl groups of the opposite configurations. This interaction was proved later by Ozaki et al. by an ultrasensitive IR spectroscopy [24,25]. These interactions result in a new arrangement of helicoidal chains (3_1_) between L- and D-lactyl units interlocked side by side within the same crystal unit (i.e., racemic crystal), indicating more dense crystal packing compared to that of the homo-crystallites. Due to the strong interactions, SC exhibits exceptional physical and chemical stabilities leading to significantly enhanced properties [23]. For example, SC crystals were found to exhibit higher melting temperatures (ca. 50 °C higher than the corresponding homo-crystals), improved mechanical properties, and stronger hydrolytic resistance compared to PLA homo-crystals. Thus, stereocomplexation between PLLA and PDLA has proven to be a powerful tool for the generation of thermally and mechanically enhanced nanomaterials.

The stereocomplexation of block copolymers containing PLLA and PDLA blocks has been applied to synthesize non-covalent ABA-type triblock copolymers for various applications (A can be crystalline or amorphous block, B is PLA stereocomplex). Several examples of the A block used in such copolymers are polyethylene glycol (PEG) [26,27,28,29], poly(*ε*-caprolactone) (PCL) [30], polymenthide (PM) [31], poly(*N*,*N*-(dimethylamino)ethyl methacrylate) (PDMAEMA) [32], polyacrylic acid (PAA) [33], polystyrene (PS) [34], and polyisoprene (PI) [35,36]. To the best of our knowledge, P2VP-*b*-PLA systems have not been used in stereocomplex systems, even though the synthesis of such diblock copolymer has been reported via the combination of ring-opening polymerization (ROP) and reversible addition–fragmentation chain-transfer (RAFT) polymerization [37,38].

While much research has been conducted on non-covalent triblock copolymers using PLA SC, little information is known about PLA SC systems containing different block copolymers, i.e., non-covalent triblock terpolymer (ABC-type triblock terpolymer). This approach provides new insights into polymer design strategies for high-performance PLA-based materials. Therefore, a fundamental study of the synthesis and properties of such materials is required to establish the structure-property relationships.

Recently, our group reported the synthesis and characterization of non-covalent PS-*b*-SC-*b*-PI triblock terpolymers by the stereocomplex formation between well-defined PI-*b*-PLLA and PS-*b*-PDLA [39]. The synthesis of these block copolymers was accomplished by combining anionic polymerization high-vacuum techniques (HVTs) and ROP. Hydroxy-terminated PI and PS were synthesized via anionic polymerization and were used as macroinitiators for the ROP of LLA and DLA in the presence of Sn(Oct)_2_ catalyst. The molecular characteristics, as well as the thermal properties of the precursors and the triblock terpolymers, were studied.

This study focuses on the synthesis and characterization of well-defined PS-*b*-SC-*b*-P2VP via the stereocomplex formation between P2VP-*b*-PLLA and PS-*b*-PDLA. Both BCPs were synthesized by combining anionic polymerization and ROP. First, hydroxy-functionalized P2VP and PS were synthesized by anionic polymerization HVTs, followed by termination with ethylene oxide and neutralization with methanol. The resulting polymers were then used as macroinitiators for the ROP of the corresponding LA using Sn(Oct)_2_ as a catalyst. The synthesized BCPs were characterized by ^1^H nuclear magnetic resonance (NMR) spectroscopy and size exclusion chromatography (SEC). Stereocomplex formation was accomplished by mixing PS-*b*-PDLA and P2VP-*b*-PLLA in chloroform, followed by precipitation in hexane. Both BCPs and their corresponding stereocomplexes were characterized by differential scanning calorimetry (DSC), Fourier-tranform infrared (FT-IR), powder X-ray diffraction (XRD), and circular dichroism (CD) spectroscopies to study the formation of PS-*b*-SC-*b*-P2VP and to evaluate the effect of the molecular weight of PLA on the resulting PS-*b*-SC-*b*-P2VP non-covalent triblock terpolymer properties.

## 2. Materials and Methods

### 2.1. For Anionic Polymerization

Benzene (VWR, Pris, France, 99%) and tetrahydrofuran (THF, VWR, Gliwice, Poland, ≥99.0%) were dried over calcium hydride (CaH_2_, 95%) followed by distillation into a glass cylinder containing polystyrilithium (PS^(−)^Li^(+)^) for benzene, and sodium/potassium alloy for THF, under high vacuum. Styrene (Sigma-Aldrich, 99%) was dried over CaH_2_ followed by distillation over di-*n*-butylmagnesium (Bu_2_Mg) and stored at −20 °C in pre-calibrated ampoules. 2-Vinylpyridine (2VP,) was dried twice over CaH_2_ and subsequently purified using a sodium mirror and triethylaluminum (TEA), followed by distillation into pre-calibrated ampoules. *sec*-Butyllithium (1.4 M in cyclohexane, Sigma-Aldrich) was diluted to the appropriate concentration in benzene for the polymerization of styrene, or in *n*-hexane (Sigma-Aldrich, 95%) for the polymerization of 2VP, and stored under vacuum at −20 °C within a home-made glass apparatus equipped with ampoules. Ethylene oxide (EO, Sigma-Aldrich, 99.5%) was purified by distillation over CaH_2_, over *n*-BuLi at 0 °C, and stored under high vacuum in ampoules. Methanol (MeOH, Sigma-Aldrich, ≥99.9%) was purified by distillation over CaH_2_ and stored in ampoules under a high vacuum.

### 2.2. For Ring-Opening Polymerization

Toluene, 1,4-dioxane (anhydrous, >99.9%), and benzoic acid (99.5%) were dried over CaH_2_ and PS^(−)^Li^(+)^. Ethyl acetate (EtOAc) was purchased from VWR Chemicals (HiPerSolv Chromanorm) and used as received. LLA (Sigma-Aldrich, Zwijndrecht, The Netherlands, 99%) and DLA (Jinan Daigang Biomaterial Co., Ltd., Jinan, China, ≥99.5%) were recrystallized from EtOAc three times and dissolved in anhydrous 1,4-dioxane, cryo-evaporating the 1,4-dioxane, followed by drying under vacuum overnight. Stannous octoate (Sn(Oct)_2_, Sigma-Aldrich, 95%) was distilled twice over anhydrous MgSO_4_ and activated 4 Å molecular sieves, followed by azeotropic distillation with dry toluene. PS-OH and P2VP-OH macronitiators obtained by anionic polymerization were dried through a freeze-drying process in benzene two times. All monomers, solvents, and catalysts for polymerizations were stored under argon (Ar) in a glove box (LABmaster SP, MBraun, Stratham, NH, USA).

### 2.3. Instrumentation

^1^H NMR measurements were performed using Bruker AVANCE III spectrometers operating at 400 or 500 MHz; chloroform-*d* (CDCl_3_, 99.8% D, Sigma-Aldrich) was used as the solvent for all samples. ^1^H NMR spectra were used to calculate the number-average molecular weight (*M*_n_) of each block by using the integrals of the characteristic signals from the end-groups and repeating unit of each block. SEC measurements were performed using Agilent SEC (Agilent Technologies, Santa Clara, CA, USA) equipped with a PLgel 5 μm MIXED-C and PLgel 5 μm MIXED-D columns. THF was used as eluent at a flow rate of 1.0 mL min^−1^ at 35 °C. The instrument was calibrated with PS standards. SEC samples were prepared by dissolving 2 mg/mL solutions in THF and filtered through 0.22 μm Teflon filters before injection. DSC measurements were performed with a Mettler Toledo DSC1/TC100 under nitrogen (N_2_) and calibrated with Indium (purity > 99.999%). The samples were first heated from 25 to 200 °C to erase the thermal history and then cooled to −20 °C with a heating/cooling rate of 10 °C min^−1^. This cycle was repeated twice before the glass transition, melting, and crystallization temperatures (*T_g_*, *T_m_*, and *T_c_*) were recorded. X-ray diffractograms were obtained from XRD Bruker D8 Advance using Cu Kα irradiation. The sample for XRD measurements was deposited on a glass substrate with an approximate size of 1.5  cm  ×  1  cm, from a chloroform solution. Circular dichroism (CD) was performed with a Jasco J-815 model, featuring a Peltier model PTC-423S/15 thermo-stabilizing system. The cell used was a 1 mm quartz suprasil cell. The solutions of the PS-*b*-PDLA, P2VP-*b*-PLLA, and their stereocomplexes were made with acetonitrile. Typical concentrations were ∼0.1 mg/mL.

### 2.4. Synthetic Procedures

The anionic polymerization of styrene and 2VP was carried out in specific custom-made glass apparatuses, which were evacuated and washed with *n*-BuLi solution prior to polymerization. Break seals were used for the introduction of reagents. Further details regarding the polymerization techniques are provided in previous reports [40,41,42].

### 2.5. Synthesis of Hydroxy-Terminated Polystyrene (PS-OH)

Styrene (5 g) was added to the appropriate amount of solvent (benzene, 5–10% polymer concentration), followed by the addition of the initiator, *sec*-BuLi (0.833 mmol). The polymerization was left to proceed until total monomer consumption (~18 h) at room temperature. An aliquot was taken to verify the molecular characteristics (molecular weight and distribution) by heat-sealing the proper constriction. EO (~1 mL) was then added to the reaction mixture and kept for 12 h at room temperature. Finally, methanol (~0.5 mL) was added for the termination of the living polymer. The polymer solution was precipitated into a large excess of methanol. The resulting polymer was filtered and dried in a vacuum oven at 40 °C for 24 h. *M*_n_ of PS-OH was calculated to be 6300 g mol^−1^ by using ^1^H NMR end-group analysis. SEC analysis indicated an *M*_n_ of 6200 g mol^−1^ and *Đ* of 1.02.

### 2.6. Synthesis of Hydroxy-Terminated Poly(2-vinylpyridine) (P2VP-OH)

2VP (6 g) was distilled into the glass reactor containing the appropriate amount of solvent (150 mL of THF), followed by the addition of *sec*-BuLi (1.2 mmol). The polymerization was conducted at −78 °C and was left to proceed for 1 h. EO (~1 mL) was then added to the reaction mixture and kept for 12 h at room temperature. Finally, methanol (~0.5 mL) was added for the termination of the living polymer. The polymer solution was precipitated into a large excess of *n*-hexane. The resulting polymer was filtered and dried in a vacuum oven at 40 °C for 24 h. *M*_n_ of P2VP-OH was calculated to be 6000 g mol^−1^ by using ^1^H NMR end-group analysis. SEC analysis indicated an *M*_n_ of 5500 g mol^−1^ and *Đ* of 1.03.

### 2.7. Synthesis of PS-b-PDLA

In a glove box under Ar atmosphere, dry PS-OH (248 mg, 0.039 mmol), Sn(Oct)_2_ (8.1 mg, 0.02 mmol), D-LA (288 mg, 2 mmol), and 3 mL dry toluene were added to a dry Schlenk flask equipped with a stirrer bar. The reaction mixture was stirred for 24 h at 80 °C, and the conversion was monitored by ^1^H NMR spectroscopy. After 24 h, the reaction mixture was quenched with benzoic acid and precipitated in cold MeOH. The resulting diblock copolymer was centrifuged and dried under a vacuum for 24 h at 40 °C.

### 2.8. Synthesis of P2VP-b-PLLA

In a glove box under Ar atmosphere, dry P2VP-OH (240 mg, 0.04 mmol), Sn(Oct)_2_ (8.1 mg, 0.02 mmol), L-LA (290 mg, 2 mmol), and 3 mL dry toluene were added to a dry Schlenk flask equipped with a stirrer bar. The reaction mixture was stirred for 24 h at 80 °C, and the conversion was monitored by ^1^H NMR spectroscopy. After 24 h, the reaction mixture was quenched with benzoic acid and precipitated in cold *n*-hexane. The resulting diblock copolymer was centrifuged and dried under vacuum for 24 h at 40 °C.

### 2.9. Stereocomplex Formation (PS-b-SC-b-P2VP)

Equimolar solutions of PS-*b*-PDLA and P2VP-*b*-PLLA were prepared separately by dissolving each polymer (~100 mg) in chloroform (5 mL) under vigorous stirring for 15 min at 400 rpm. Subsequently, the two solutions were mixed and stirred for another 15 min at 400 rpm. Finally, the final solution was precipitated in cold n-hexane (200 mL) and stirred for 30 min at 200 rpm. The precipitate was centrifuged and dried under vacuum for 24 h at 40 °C. In the following discussion, the stereocomplex-based samples (PS-b-SC-b-P2VP) are referred to as SCPLA_x_, where x is the calculated molecular weight of the PLA segments.

## 3. Results and Discussion

**Scheme 1:**
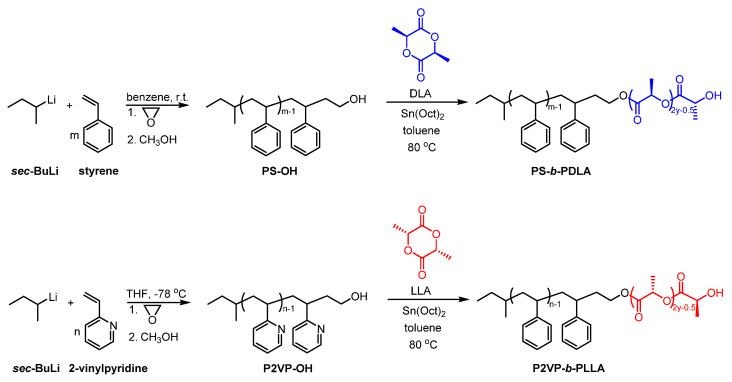
General reactions for the synthesis of ω-hydroxyl functionalized PS and P2VP via anionic polymerization and the subsequent ROP of DLA/LLA.

The anionic polymerization of 2VP and styrene was carried out in THF (at −78 °C) and benzene (at room temperature), respectively, using *sec*-BuLi as the initiator (Scheme 1). After complete consumption of monomers, the living polymers were end-capped by an excess amount of EO at room temperature. Quantitative functionalization reaction of polymeric organolithium compounds in hydrocarbon solutions with EO at room temperature proceeds in the absence of EO oligomerization [43]. The anionic polymerization of EO does not happen under these conditions, resulting in initiation without propagation. Therefore, only one monomeric unit of EO is inserted at the chain-end. This is due to the high charge density of the lithium cation resulting in the strong aggregation of terminal lithium alkoxides.

The functionalization of both polymers (PS and P2VP) with EO was confirmed by ^1^H NMR and by FT-IR spectrosocopies. ^1^H NMR spectrum shows a peak around (*δ* = 3.2–3.7 ppm), which corresponds to the –CH_2_ attached to the hydroxyl end-group (Figure S1). Moreover, the –OH group can be observed using FT-IR as a broad peak around 3401 cm^−1^ and 3394 cm^−1^ for PS-OH and P2VP-OH, respectively (Figure S2). Further confirmation is evident by the successful copolymerization of PLA via ROP using PS-OH and P2VP-OH as macroinitiators, as confirmed by SEC and ^1^H NMR spectroscopy (Table 1).

PS-OH and P2VP-OH were synthesized with an *M*_n_ of 6300 and 6000 g mol^−1^, as obtained by ^1^H NMR end-group analysis, respectively. Their molecular characteristics are presented in Table 1. Both homopolymers have low molar-mass dispersity, as indicated by SEC, suggesting that the polymers can be considered to be well defined (Figure S3).

ROP of DLA/LLA initiated by dry PS-OH and P2VP-OH macroinitiators and catalyzed by Sn(Oct)_2_ was performed in toluene at 80 °C to afford PS-*b*-PDLA and P2VP-*b*-PLLA diblock copolymers, respectively. Sn(Oct)_2_, is considered one of the most effective catalysts for the ROP of lactides under a wide range of conditions [44,45,46,47,48]. Moreover, it is commercially available, soluble in most organic solvents, and has been approved by the United States Food and Drug Administration. The targeted molecular weights of PLAs were varied: 5000, 7000, and 10,000 g mol^−^^1^. The molecular characteristics of the resulting diblock copolymers were determined by SEC and ^1^H NMR measurements and are presented in Table 1.

**Figure 1:**
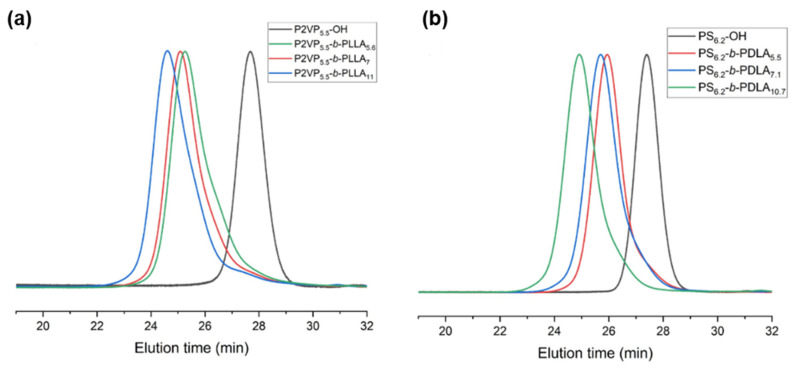
SEC traces of (**a**) P2VP-OH precursor and P2VP-*b*-PLLA diblock copolymers and (**b**) PS-OH precursor and PS-*b*-PDLA diblock copolymers (THF as eluent, 35 °C, PS standards).

Figure 1 shows the SEC traces of the homopolymer precursors compared to the corresponding diblock copolymers. The SEC traces clearly show a shift towards a lower elution time, indicating an increase in molecular weight compared to the PS-OH and P2VP-OH precursors. The *Đ* values of all copolymers are below 1.1 (between 1.02 and 1.08), indicating that the diblock copolymers are nearly uniform (in molar mass).

**Figure 2:**
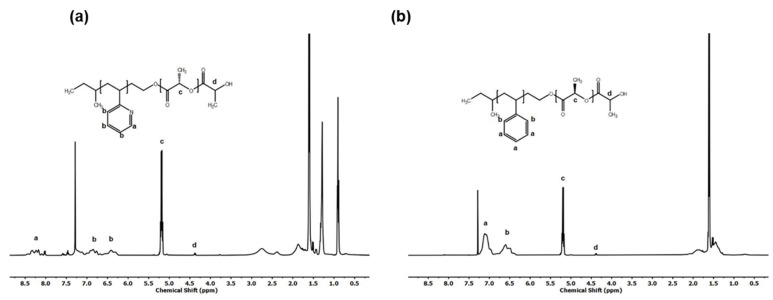
^1^H NMR spectra of (**a**) P2VP_5.5_-*b*-PLLA_5.6_, and (**b**) PS_6.2_-*b*-PDLA_5.5_ diblock copolymers (400 MHz, CDCl_3_, 25 °C).

For the following discussion on ^1^H NMR, FT-IR, and CD results, P2VP_5.5_-*b*-PLLA_5.6_ and PS_6.2_-*b*-PDLA_5.5_ will be used as representative samples. ^1^H NMR spectra of the diblock copolymers (Figure 2) show the characteristic peaks of methine proton from PLA main chain (c, *δ* = 5.2–5.3 ppm) and the terminal C–H (d, *δ* = 4.3–4.4 ppm). The molecular weights of the PLA blocks were determined by calculating the integral ratio of proton (c) and (d), i.e., end-group analysis.

**Figure 3:**
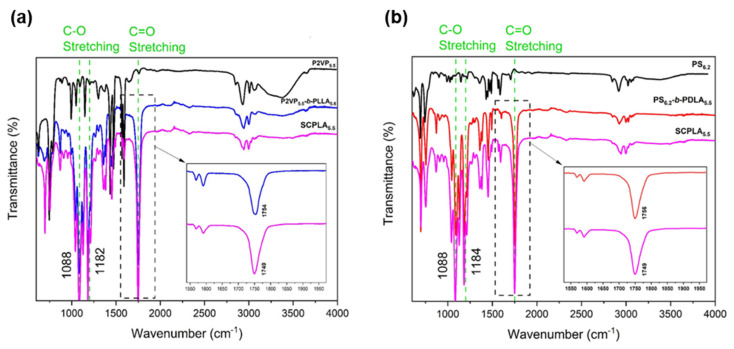
FT-IR spectra of (**a**) P2VP_5.5_-OH, P2VP_5.5_-*b*-PLLA_5.6_, and SCPLA_5.5,_ and (**b**) PS_6.2_-OH, PS_6.2_-*b*-PDLA_5.5_ and SCPLA_5.5_.

FT-IR spectroscopy was used to investigate the formation of the diblock copolymers and their corresponding stereocomplexes. The FT-IR spectra of the diblock copolymers reveal that a new peak is present at ∼1750 cm^−1,^ which corresponds to the carbonyl (C=O) stretching, and two other peaks at ∼1184 and 1088 cm^−1^ correspond to the (C–O) stretching of PLA (Figure 3).

Upon the formation of stereocomplex, the vibrational stretch of the carbonyl group of PLA, i.e., ν(C=O) band, in the SCPLA (Figure 3) shifted to a slightly lower wavenumber than that of the PS-*b*-PDLA (from 1756 to 1749 cm^−1^) and P2VP-*b*-PLLA (from 1754 to 1749 cm^−1^). This shift is attributed to the arrangement of the PLA chains into a more dense crystal packing due to stereocomplex formation via intermolecular H-bond interaction [49].

The specific optical rotation of PDLA/PLLA blocks in the block copolymers was evaluated by CD experiments. It is worth noting that PDLA chains take the right-handed helical conformation, whereas PLLA takes the left-handed helical conformation in acetonitrile solution. Figure S4 shows the CD spectra for both PS_6.2_-*b*-PDLA_5.5_ and P2VP_5.5_-*b*-PLLA_5.6_ in acetonitrile. The carboxylic group of PLAs with a helical conformation is accompanied by a characteristic absorption band of n → π* transition. Therefore, a positive Cotton effect for P2VP_5.5_-*b*-PLLA_5.6_ and a negative Cotton effect for PS_6.2_-*b*-PDLA_5.5_ can be observed at ~233 nm. On the other hand, the solution of SCPLA_5.5_ does not show such an effect, indicating that the D- and L-helical conformations complement each other due to the stereocomplex formation, resulting in zero CD response.

**Figure 4:**
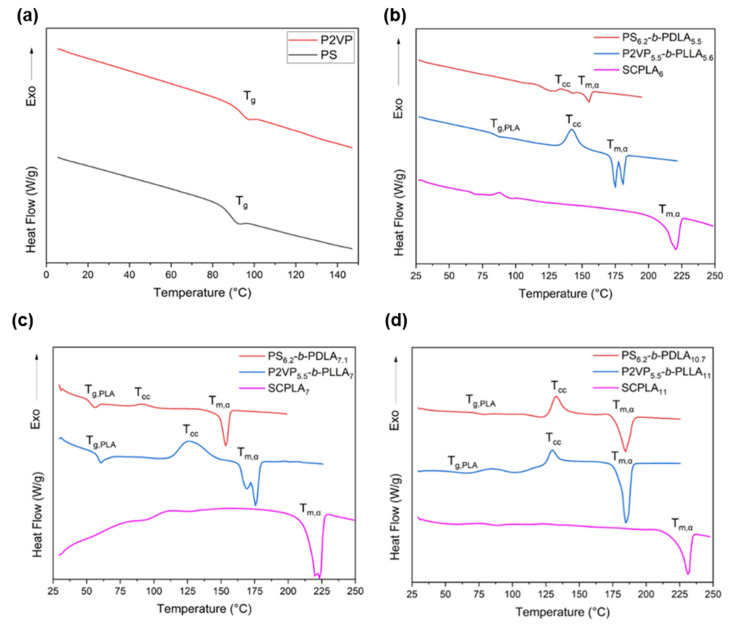
DSC thermograms of PS, P2VP homopolymers (**a**), PS-*b*-PDLAs, P2VP-*b*-PLLAs, and the corresponding SCPLAs with varying PLA segments with molecular weights of (**b**) 5 kg mol^−1^ (**c**) 7 kg mol^−1^, and (**d**) 11 kg mol^−1^ (heating scan 10 °C/min, under N_2_ atmosphere).

**Figure 5:**
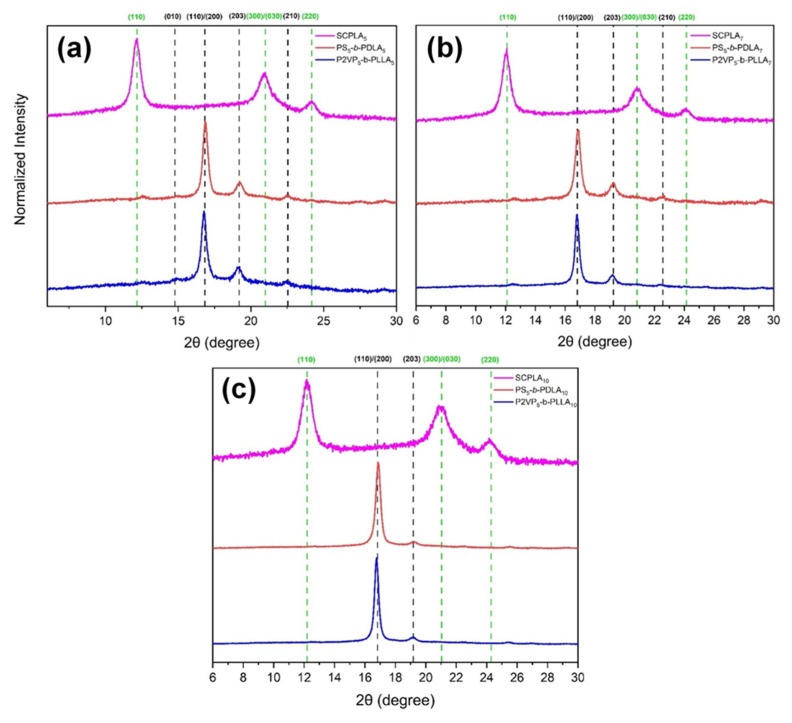
XRD patterns of PS-*b*-PDLAs, P2VP-*b*-PLLAs, and the corresponding SCPLAs with PLA segments with molecular weight of (**a**) 5 kg mol^−1^, (**b**) 7 kg mol^−1^, and (**c**) 11 kg mol^−1^ (samples were cast on top of the glass substrate).

The influence of the molecular weight of PLA segments on the physical properties of the diblock copolymers, as well as their corresponding stereocomplexes, was investigated on the basis of DSC and XRD analyses. Figure 4 and Figure 5 show the DSC thermograms and XRD patterns of the block copolymers and the stereocomplexes obtained by precipitation.

DSC analysis was performed in order to evaluate the thermal properties, including glass transition temperature (*T_g_*) and melting temperature (*T_m_*), of the homopolymers (PS_6.2_-OH and P2VP_5.2_-OH) and diblock copolymers (PS_6.2_-*b*-PDLA_x_ and P2VP_5.2_-*b*-PLLA_y_), as well as the corresponding SCPLAs. The DSC thermograms of the homopolymers, block copolymers, and corresponding SCPLAs are shown in Figure 4.

The *T_g_* values of the amorphous PS_6.2_-OH and P2VP_5.2_-OH precursors are observed to be 92.3 °C and 93.6 °C (Table 2), respectively. In the case of the diblock copolymers, the *T_g_* values of PS and P2VP blocks cannot be observed, indicating that the PS/P2VP (amorphous blocks) and PLLA/PDLA (crystalline blocks) are miscible in the melt [50,51]. The PLLA/PDLA crystallites are well organized (as proved by the distinct Δ*H*_m_) and limit the mobility of the amorphous blocks [50]. In addition, for PS_6.2_-*b*-PDLA_7.1_ (Figure 4c), the *T_g_* of PS overlaps with the exothermic peaks from the cold-crystallization temperature (*T_cc_*) of PLA. Therefore, the effect of the molecular weight of crystalline PLA blocks on the *T_g_* of the amorphous blocks cannot be evaluated. The small endothermic humps observed between 50 and 80 °C are attributed to the *T_g_* values of PLA.

All block copolymers exhibited a *T_m_* of PLAs in the range of 150–180 °C, indicating the existence of crystalline PDLA/PLLA block. The PLLA block in P2VP_5.2_-*b*-PLLA_5.6_ and P2VP_5.2_-*b*-PLLA_7_ shows double melting (*T_m_*) peaks. Two plausible explanations have been proposed for this observed phenomenon. The first concerns the lamellar crystal thickness [52,53], and suggests that the double endothermic behavior is the result of the existence of two kinds of crystal lamellae having different thicknesses. Consequently, the melting of the thinner lamellae would be observed at a lower temperature endotherm, whereas the thicker lamellae are related to the higher temperature endotherm. The second possible explanation is the partial melting and recrystallization process [54,55], where the lower-temperature endotherm is the result of the melting of the initial lamellae followed by recrystallization into more perfect lamellae.

In general, the *T_m_* values of PLLA and PDLA are affected by the increase in molecular weight. Such a trend is also observed in the PS_6.2_-*b*-PDLA and P2VP_5.2_-*b*-PLLA block copolymers. The higher the molecular weight of PLLA and PDLA, the higher the *T_m_* in the diblock copolymers. When the molecular weight is increased from ~5000 g mol^−1^ to ~10,000 g mol^−1^, the *T_m_* values increase from 153.0 to 162.6 °C for PDLA-containing BCPs, and from 156.0 to 162.9 °C in the case of PLLA-containing BCPs. Similarly, the *T_m_* and the melting enthalpy (∆*H_m_*) of SCPLA also increase with the increase in the molecular weight of PLA (from 220.3 to 230.3 °C). These results are in good agreement with our recent findings on the thermal properties of PS-*b*-SC-*b*-PI [39]. It is worth noting that the *T_m_* of SCPLA is always ~70 °C higher than the *T_m_* of their corresponding diblock copolymers, indicating that the effect of the amorphous PS_6.2_ and P2VP_5.2_ on the crystal packing of SCPLA is not significant in this case.

The investigation of crystal structure and degree of crystallinity (*X_c_*) for the homopolymers, block copolymers, and their stereocomplexes obtained by precipitation was carried out by means of XRD analysis. The diffraction patterns are presented in Figure 5. The diblock copolymers exhibited diffraction peaks at 2*θ* = 16.7°, 17.6°, 19.5°, 22°, and 26°, verifying the presence of *α* crystals, i.e., orthorhombic unit cells. In addition, the crystal structure of SCPLAs obtained from the equimolar ratio of PLLA:PDLA was also investigated. The diffractograms (Figure 5) show diffraction peaks of triclinic crystal at 2*θ* = 14°, 24°, and 28°, confirming the formation of stereocomplexes. Overall, the XRD patterns of block copolymers and their stereocomplexes are in good agreement with the literature, as the *α* crystals show the reflection at 2*θ* = 16.6°, 19.1° and 17°, 19°, and the SCPLA crystals show the reflection at 2*θ* = 12°, 21°, 24°, and 12°, 20.9°, 24° [23].

The total degree of crystallinity (*X_c_*) of a polymeric material can be calculated from its XRD pattern. *X_c_* is defined as the ratio of the area of all crystalline peaks to the total area under the XRD peaks (crystalline + amorphous), as shown in Equation (1):

(1) $$\%\;Crystallinity=\frac{I_{c}}{\left(I_{c}+I_{a}\right)}\times 100,$$

where *I_a_* and *I_c_* are the areas of the amorphous and crystalline domains, respectively. Based on DSC and XRD results, both PS and P2VP segments are amorphous. Therefore, the *X_c_* of the diblock copolymers and their SCPLAs obtained from XRD can be attributed to the *X_c_* of their PLA segments.

As can be seen in Figure 5, the *X_c_*s of PS-*b*-PDLAs (14.5 < *X_c_* < 52.4) and P2VP-*b*-PLLAs (34.0 < *X_c_* < 51.4) increase with increasing molecular weight of the PLA blocks. This clearly indicates that the fraction crystalline domain increases with increasing molecular weight of PLA. A similar trend is also observed for the SCPLA (33.0 < *X_c_* < 39.3), although the increment is insignificant.

## 4. Conclusions

Several well-defined diblock copolymers, PS_6.2_-*b*-PDLA_x_ and P2VP_5.5_-*b*-PLLA_y_, were successfully synthesized via the combination of anionic polymerization high-vacuum techniques and ring-opening polymerization. PS_6.2_-*b*-PDLA_x_ and P2VP_5.5_-*b*-PLLA_y_ were used as the precursors to synthesize non-covalent PS_6.2_-*b*-SC-*b*-P2VP_5.5_ triblock terpolymers via stereocomplexation of PDLA and PLLA blocks in chloroform. ^1^H NMR spectroscopy and SEC confirmed the molecular characteristics of the copolymers. FT-IR, DSC, XRD, and CD spectroscopies revealed the formation of PS_6.2_-*b*-SC-*b*-P2VP_5.5_ as well as the effect of varying PLA molecular weights on the thermal properties of co/terpolymers. It was found that the *T_m_* and *X_c_* of the co/terpolymers increase with the increase of the molecular weights of PLA segments.

Comprehensive studies are necessary to further understand this system and determine a range of potential applications. Morphological and mechanical studies, including Young’s modulus and tensile strength, will be conducted to fully establish the structure–properties relationship of these new non-covalent triblock terpolymers. Moreover, the presence of P2VP segments in these triblock terpolymers can be promising for biomedical applications due to their pH-sensitive nature and the ability to bind with metal cations.

## Figures and Tables

**Table 1:** Molecular characteristics of homopolymers, diblock copolymers, and the corresponding stereocomplex.

Entry	Sample	Conv ^c^ (%)	M_n_ (kg mol^−1^)	*Đ* ^d^
1	PS_6.2_-OH ^a^	100	6.20 ^d^	1.02
2	P2VP_5.5_-OH ^a^	100	5.54 ^d^	1.03
3	PS_6.2_-*b*-PDLA_5.5_^b^	97	5.49 ^e^	1.03
4	P2VP_5.5_-*b*-PLLA_5.6_^b^	96	5.57 ^e^	1.08
5	PS_6.2_-*b*-PDLA_7.1_ ^b^	98	7.06 ^e^	1.04
6	P2VP_5.5_-*b*-PLLA_7_ ^b^	99	6.96 ^e^	1.04
7	PS_6.2_-*b*-PDLA_10.7_ ^b^	99	10.7 ^e^	1.05
8	P2VP_5.5_-*b*-PLLA_11_ ^b^	99	11.0 ^e^	1.07

^a^ Synthesized by anionic polymerization high-vacuum techniques. ^b^ Synthesized by ROP of DLA/LLA with Sn(Oct)_2_ as the catalyst. ^c^ Conversions of the monomers were determined by 400 MHz ^1^H NMR spectra of crude products in CDCl_3_ at 25 °C. ^d^ Determined by SEC in THF at 35 °C (calibrated with PS standards). ^e^ The molecular weight corresponds to the PLA block.

**Table 2:** Thermal properties of homopolymers, block copolymers, and the corresponding SCPLA.

Sample	*T_g_* (°C) ^1^	*T_m_* (°C) ^1^	∆*H_m_* (J/g) ^1^	*X_c_* (%) ^2^
PS_6.2_-OH	92.3	-	-	Amorphous
P2VP_5.5_-OH	93.6	-	-	Amorphous
PS_6.2_-*b*-PDLA_5.5_	-	153.0	24.4	14.5
P2VP_5.5_-*b*-PLLA_5.6_	83.0	156.0	23.5	34.0
SCPLA_5.5_	-	220.3	36.1	33.0
PS_6.2_-*b*-PDLA_7.1_	54.2	154.0	25.5	37.3
P2VP_5.5_-*b*-PLLA_7_	58.9	152.6	27.7	39.6
SCPLA_7_	-	223.3	41.3	38.3
PS_6.2_-*b*-PDLA_10.7_	73.9	162.6	30.0	52.4
P2VP_5.5_-*b*-PLLA_11_	59.9	162.9	43.2	51.4
SCPLA_11_	-	231.1	39.9	39.3

^1^ Determined by DSC (heating scan 10 °C min^−1^, N_2_ atmosphere). ^2^ Determined by XRD (samples were deposited on top of a glass substrate).

## Data Availability

Not applicable.

## Supplementary Materials

The following supporting information can be downloaded at: https://www.mdpi.com/article/10.3390/polym14122431/s1. Figure S1: ^1^H NMR (400 MHz, CDCl_3_) spectra of (a) PS-OH and (b) P2VP-OH; Figure S2: FT-IR spectra of (a) PS-OH and (b) P2VP-OH; Figure S3: SEC traces of (a) PS-OH and (b) P2VP-OH in THF at 35 °C; Figure S4: CD spectra of PS_6.2_-b-PDLA_5.5_, P2VP_5.5_-b-PLLA_5.6_, and SCPLA_5.5_ were measured in acetonitrile with a concentration of 0.1 mg mL^−1^ at room temperature.
