# Supplementary material for: Synthesis and Thermal Analysis of Non-Covalent PS-b-SC-b-P2VP Triblock Terpolymers via Polylactide Stereocomplexation
Source: Polymers (Basel). 2022 Jun 15;14(12):2431. doi: 10.3390/polym14122431 (PMC9228810; doi:10.3390/polym14122431)
Supplement: Supplementary file 1 [file polymers-14-02431-s001.zip › polymers-1761225-supplementary.pdf]

Synthesis and Thermal Analysis of Non-Covalent PS-*b*-SC-*b*-P2VP Triblock Terpolymer *via* Polylactide Stereocomplexation

Ameen Arkanji, Viko Ladelta*, Konstantinos Ntetsikas*, Nikos Hadjichristidis*

Polymer Synthesis Laboratory, KAUST Catalysis Center, Physical Sciences and Engineering Division, King Abdullah University of Science and Technology (KAUST), Thuwal 23955, Saudi Arabia.

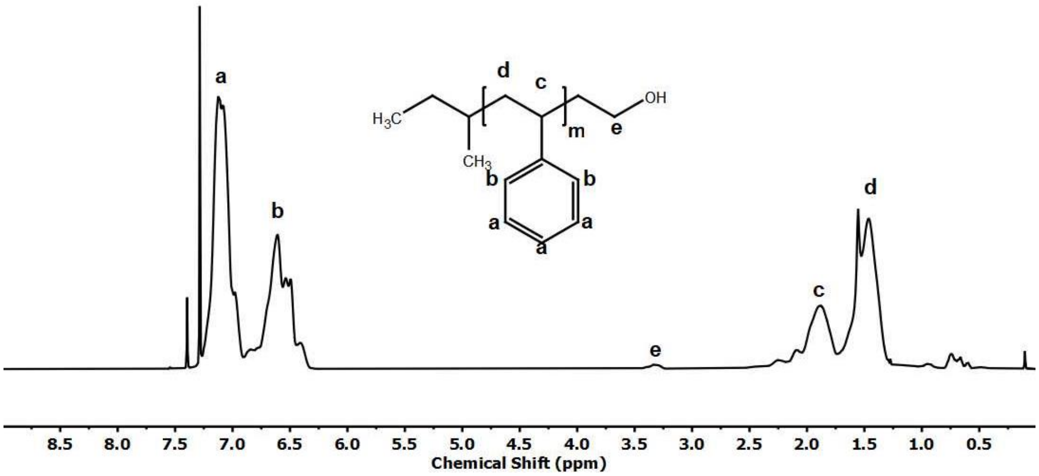

(**a**)

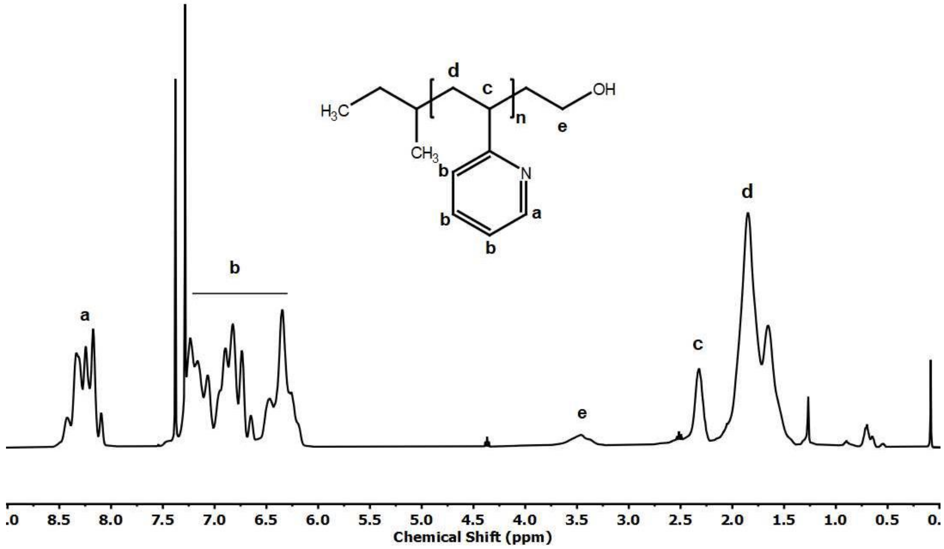

(**b**)

Figure S1. ^{1}H NMR (400 MHz, $CDCl_3$) spectra of **(a)** PS-OH and **(b)** P2VP-OH.

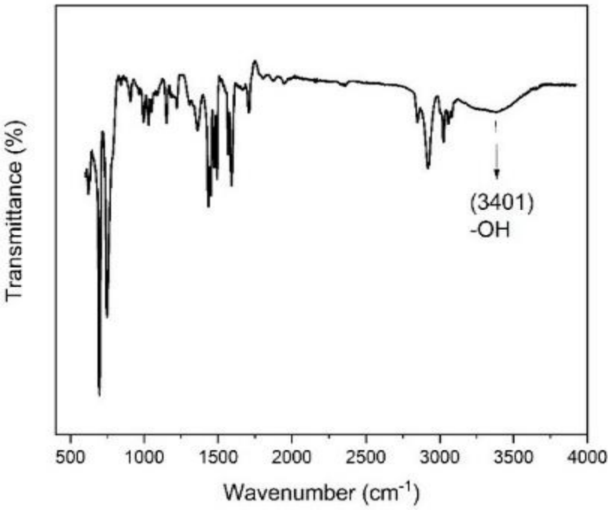

(**a**)

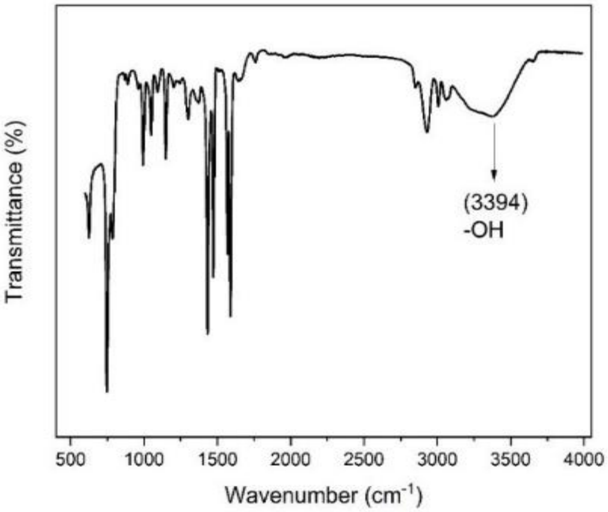

(**b**)

Figure S2. FT-IR spectra of **(a)** PS-OH and **(b)** P2VP-OH.

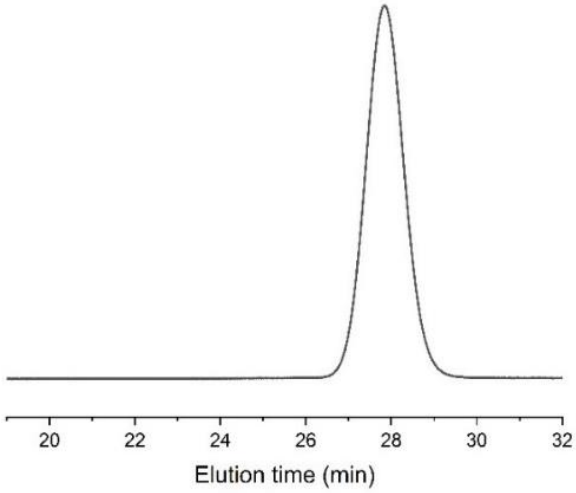

(**a**)

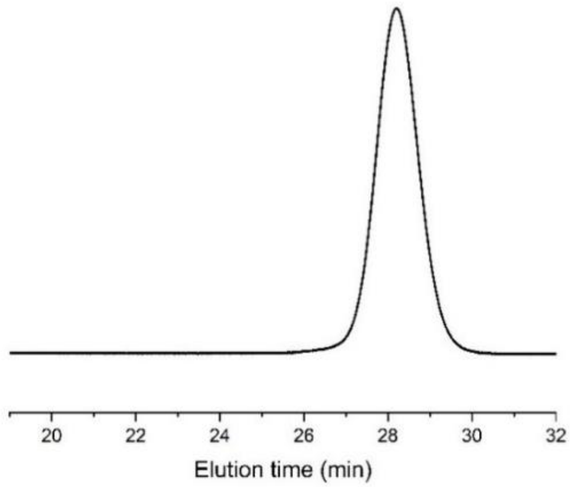

(**b**)

Figure S3. SEC traces of **(a)** PS-OH and **(b)** P2VP-OH in THF at 35 °C.

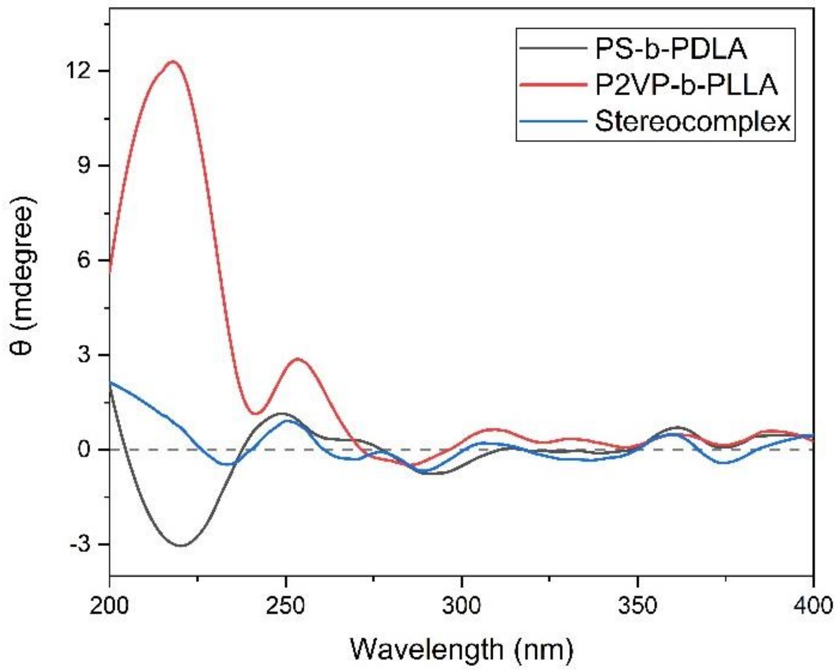

Figure S4. CD spectra of $PS_{6.2}$-*b*-$PDLA_{5.5}$, $P2VP_{5.5}$-*b*-$PLLA_{5.6}$, and $SCPLA_{5.5}$ were measured in acetonitrile with a concentration of 0.1 mg mL^{-1} at room temperature.
